# Prognostic significance of GAD1 overexpression in patients with resected lung adenocarcinoma

**DOI:** 10.1002/cam4.2345

**Published:** 2019-06-17

**Authors:** Mitsuhiro Tsuboi, Kazuya Kondo, Kiyoshi Masuda, Shoichiro Tange, Koichiro Kajiura, Tomohiro Kohmoto, Hiromitsu Takizawa, Issei Imoto, Akira Tangoku

**Affiliations:** ^1^ Department of Thoracic, Endocrine Surgery and Oncology, Institute of Biomedical Sciences Tokushima University Graduate School Tokushima Japan; ^2^ Department of Oncological Medical Services, Institute of Biomedical Sciences Tokushima University Graduate School Tokushima Japan; ^3^ Department of Human Genetics, Institute of Biomedical Sciences Tokushima University Graduate School Tokushima Japan; ^4^ Kawasaki Medical School Kurashiki Japan; ^5^ Division of Molecular Genetics Aichi Cancer Center Research Institute Nagoya Japan; ^6^ Department of Cancer Genetics Nagoya University Graduate School of Medicine Nagoya Japan

**Keywords:** DNA methylation, expression, GAD1, lung adenocarcinoma, prognosis

## Abstract

**Background and Objectives:**

In a previous genome‐wide screening, we identified hypermethylated CpG islands around *glutamate decarboxylase 1* (*GAD1*) in lung adenocarcinoma (LADC). In this study, we aimed to investigate the methylation and expression status of GAD1 and its prognostic value in patients with LADC.

**Methods:**

*GAD1* methylation and mRNA expression status were analyzed using 33 tumorous and paired non‐tumorous LADC samples and publicly available datasets. The prognostic value of GAD1 overexpression was investigated using publicly available datasets of mRNA levels and 162 cases of LADC by immunohistochemistry.

**Results:**

The methylation and mRNA expression levels of GAD1, each having a positive correlation, were significantly higher in LADC tumors than in paired non‐tumorous tissues. LADC patients with higher GAD1 mRNA expression showed significantly poorer prognosis for overall survival in publicly available datasets. Higher immunoreactivity of GAD1 was significantly associated with the pathological stage, pleural invasion, lymph vessel invasion, and poorer prognosis for cancer‐specific and disease‐free survival. Multivariate analysis revealed that GAD1 protein overexpression is an independent prognosticator for disease‐free survival.

**Conclusions:**

GAD1 mRNA and protein expression levels were significant prognostic factors in LADC, suggesting that they might be useful biomarkers to stratify patients with worse clinical outcomes after resection.

## 1 INTRODUCTION

Lung adenocarcinoma (LADC) is the predominant histological subtype of lung cancer and has the highest mortality rate worldwide.^1^, ^2^ Although progress in the treatment of LADC has improved short‐term survival, the impacts on long‐term survival remain modest.^3^ Therefore, a better understanding of the mechanisms of LADC tumor progression is needed and useful prognostic molecular markers for accurately predicting the clinical outcomes of LADC are of great clinical significance.

To identify genes in the tumor that are specifically methylated at an early‐stage of LADC, we had previously performed a genome‐wide screening of aberrantly methylated CpG islands (CGIs) using paired tumorous and non‐tumorous tissues of early‐stage LADC, and identified *TRIM58* as a novel candidate tumor‐suppressor gene for this disease.^4^ Through this screening, the glutamate decarboxylase 1 gene (*GAD1*) was found to be nearby hypermethylated CGIs in LADC. Because paradoxical hypermethylation‐associated overexpression of *GAD1* was reported recently in colorectal and liver cancers^5^ and *GAD1* overexpression has been reported in various neoplastic tissues, such as oral, nasopharyngeal, colorectal, liver, and gastric cancers,^5^, ^6^, ^7^, ^8^, ^9^ we focused on *GAD1* as a potential LADC‐related gene in the present study. Moreover, the methylation and expression status and clinicopathological significance of *GAD1* in LADC tumorigenesis have also not been examined previously.

Therefore, in the present study, we investigated the DNA methylation and mRNA and protein expression status of GAD1 in resected LADC tumors. Moreover, we assessed the prognostic significance of GAD1 expression in LADC using our tumor panel and publicly available datasets.

## 2 MATERIALS AND METHODS

### 2.1 Selection of candidate CGI

Previously obtained Human Methylation 450K array‐based methylation screening data of 12 paired tumorous/non‐tumorous stage‐I LADC sample sets from patients (6 smokers and 6 never‐smokers) who underwent surgery at Tokushima University Hospital (Tokushima, Japan) between April 1999 and March 2015 were reevaluated (Table S1).^4^

### 2.2 Patients and tissue samples

We included tumors and non‐tumorous tissues of LADC that were surgically resected at Tokushima University Hospital between April 1999 and November 2013 for additional analyses. No patients had been administered preoperative radiation, chemotherapy, or immunotherapy. For pyrosequencing‐based methylation analysis and real‐time PCR‐based expression analysis, 33 LADC samples were used (Table S2). For immunohistochemical staining, 162 LADC samples were used (Table S3). The mean follow‐up duration for the 162 patients with LADC was 48 months (range, 0.6‐147 months), with 45 recurrences (27.8%) and 34 deaths (21.0%) among the patients. Tumor staging was determined based on the seventh tumor‐node‐metastasis (TNM) classification for lung cancer.^10^ The tumors were classified according to the predominant histological subtype, as proposed by the 2015 WHO classification.^11^

This study was performed in accordance with the principles outlined in the Declaration of Helsinki. The ethics committee of Tokushima University Hospital approved the study (approval number 3048), and formal written consent was obtained from all patients or their representatives.

### 2.3 DNA and RNA preparation and bisulfite conversion of genomic DNA

DNA and RNA were extracted using standard methods. Bisulfite conversion of DNA was conducted using the EpiTect Bisulfite Kit (QIAGEN GmbH, Hilden, Germany) following the manufacturer's instructions.

### 2.4 Bisulfite pyrosequencing

Bisulfite‐treated genomic DNA was amplified using a set of primers designed with PyroMark Assay Design Software version 2.0.01.15 (QIAGEN GmbH, Table S4). The target region for sequencing began 10 nucleotides (nt) before and ended 26 nt after cg15126544. PCR product pyrosequencing and methylation quantification were performed with sequencing primers using the PyroMark 24 Pyrosequencing System, version 2.0.6 (QIAGEN GmbH), according to the manufacturer's instructions.

### 2.5 Real‐time quantitative reverse‐transcription polymerase chain reaction (rqRT‐PCR)

Complementary DNA was generated from isolated total RNA using the PrimeScript II first strand cDNA Synthesis Kit (TaKaRa, Shiga, Japan). rqRT‐PCR was performed using KAPA PROBE FAST qPCR Kits (Kapa Biosystems, Wilmington, MA, USA) and TaqMan Gene Expression Assays (Thermo Fisher Scientific, Waltham, MA, USA; Table S4) according to the manufacturers’ instructions. *GAPDH* mRNA levels were used as internal controls for normalization. Relative expression of *GAD1* mRNA was calculated using Human Lung Total RNA (TaKaRa) as a normal lung control.

### 2.6 Data mining in bioinformatics

Available RNA sequencing data (IlluminaHiSeq_RNASeqV2 Level 3) containing 488 tumor and 58 non‐tumor samples and Infinium Human Methylation 450K data (Level 3) containing 473 tumor and 32 nontumorous samples of LADC cases with clinical annotations were downloaded from The Cancer Genome Atlas (TCGA) Research Network (http://cancergenome.nih.gov). mRNA expression data and DNA methylation data were available for 36 and 29 paired tumorous/non‐tumorous sample sets, respectively; both types of data were available for 18 sets. Tumorous samples with mRNA expression data and survival data were available for 423 cases. Survival analyses were conducted on patients with normalized mRNA expression and overall survival (OS) profiles. Patients were divided into low‐ and high‐expression groups according to the median *GAD1* mRNA expression value.

Kaplan‐Meier Plotter (KM plotter, http://kmplot.com/analysis/), a publicly available online database of published microarray datasets for primary tumors with clinical information,^12^ was also used to generate OS curves in 9 studies from Gene Expression Omnibus (GEO, https://www.ncbi.nlm.nih.gov/geo/, Table S5) by setting the auto‐selected best value of *GAD1* mRNA expression as the cutoff. All other parameters were left at default settings.

### 2.7 Immunohistochemical staining

Paraffin sections (4‐µm thick) were subjected to immunohistochemical staining using the Envision system (ChemMate Envision kit; Dako, Glostrup, Denmark) according to the manufacturer's instructions. Antigen retrieval was performed by heating the dewaxed and dehydrated sections in Dako Real Target Retrieval Solution, pH 9 (Dako), using a 2100 retriever (Aptum Biologics, Ltd., Southampton, UK). A mouse anti‐GAD67 monoclonal antibody (Sigma‐Aldrich, St. Louis, MO, USA; G5419), diluted to 1:200 with antibody diluents (Dako), was used as the primary antibody. The proportion and intensity of GAD1 staining in the LADC samples were scored (Table S6A) independently by two different researchers.

### 2.8 Statistical analysis

Student's *t* test or Fischer's exact test was used for comparisons between two groups. The paired *t* test was used for comparisons between paired samples. The relationship between continuous variables was investigated by calculating the Spearman's correlation coefficient. For survival analysis, Kaplan‐Meier survival curves were constructed for groups based on univariate predictors, and differences among groups were tested with the log‐rank test. Univariate and multivariate survival analyses were performed using the likelihood ratio test of the stratified Cox proportional hazard regression analysis. Differences were assessed using two‐sided tests and were considered significant at a *P* < 0.05. Statistical analyses were performed using IBM SPSS version 24 (IBM Corporation, Armonk, NY) or the Survival package for R (https://cran.r-project.org).

## 3 RESULTS

### 3.1 Methylation status of CGIs and each CpG site within CGIs around GAD1

**Figure 1:**
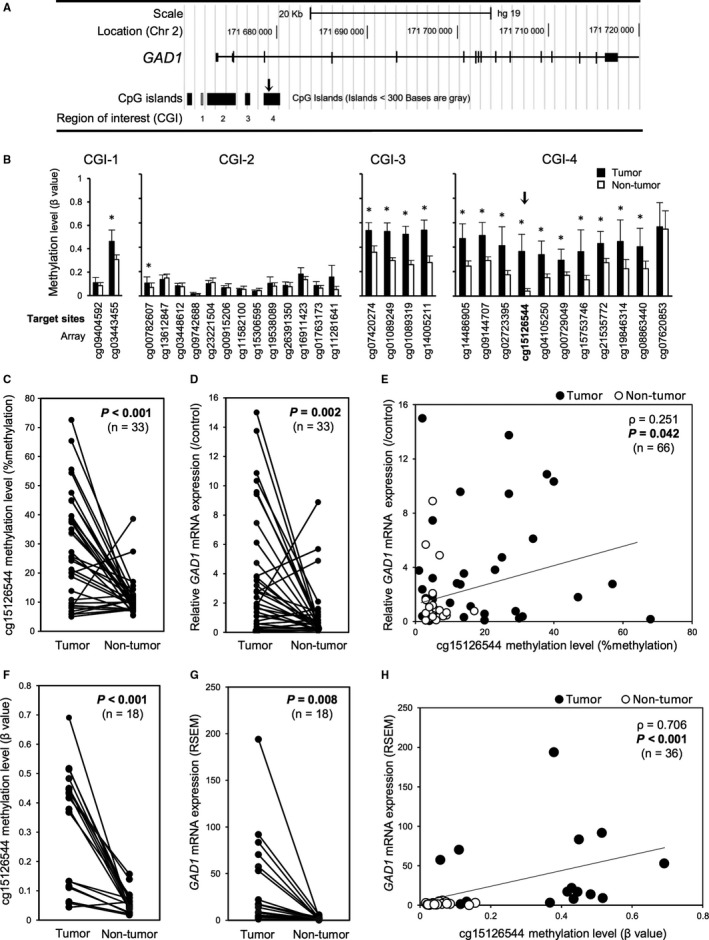
DNA methylation and mRNA expression status of *GAD1* in patients with LADC. (A) A schematic diagram of the *GAD1* gene structure with CGIs around *GAD1*. The arrow indicates the location of cg15126544. (B) The average *β*‐value (methylation level) of each CpG site targeted in the array‐based methylation experiment involving 12 LADC cases. *P < 0.05 vs. paired non‐tumorous tissues. (C) Linear plots of the average DNA methylation values (percentages) of cg15126544 in 33 LADC tumorous and paired non‐tumorous tissues, as determined by quantitative pyrosequencing. Samples from the same patient are linked with straight lines. (D) Linear plots of expression levels of *GAD1* mRNA relative to those of the control normal human lung in 33 LADC tumorous and paired non‐tumorous tissues. Relative expression of *GAD1* mRNA was calculated using Human Lung Total RNA as a normal control. (E) Correlation between the average methylation levels of cg15126544 (*x*‐axis) and relative *GAD1* mRNA expression levels (*y*‐axis) in 33 LADC tumorous and paired non‐tumorous tissues. (F) Linear plots of the methylation levels (*β*‐values) of cg15126544 determined through an array‐based methylation experiment using HumanMethylation450K array in 18 paired LADC tumor and non‐tumorous tissue samples obtained from the TCGA dataset (http://cancergenome.nih.gov). (G) Linear plots of mRNA expression of *GAD1* determined by RNA sequencing and quantified by RNA‐Seq by Expectation Maximization (RSEM) in 18 paired LADC tumor and non‐tumorous tissue samples obtained from the TCGA dataset. (H) Correlation between the methylation levels (*β*‐values) of cg15126544 (*x*‐axis) and *GAD1* mRNA expression levels (*y*‐axis) in 18 paired LADC tumor and non‐tumorous tissue samples obtained from the TCGA dataset

In a previous array‐based, genome‐wide methylation screening of 12 paired tumorous/non‐tumorous LADC sample sets,^4^ CGI‐3 around *GAD1* was ranked 14th as a hypermethylated CGI with a high *P*‐value (Table S1). Because hypermethylation‐associated overexpression of *GAD1* was reported in colorectal and liver cancers,^5^ we reevaluated the results of the array‐based methylation status of each CpG site within CGI‐1‐4 (Figure 1A) around *GAD1* (Figure 1B). The methylation levels of all CpG sites determined by array‐based analysis within CGI‐3 and in tumors were significantly higher than those in paired non‐tumorous tissues. Although the methylation levels in tumors were higher in CpG sites within CGI‐3 than in those within CGI‐4, the average *β*‐value in non‐tumor tissues was extremely and specifically low at cg15126544 and showed the largest difference of average *β*‐value between tumor and non‐tumor tissues at this site (Figure 1B and Table S7), which is localized within the CCCTC‐binding factor (CTCF)‐binding site of *GAD1*. Similar results were observed in the Level 3 Infinium Human Methylation 450K data of 29 LADC tumors and paired non‐tumor tissues from TCGA dataset (Figure S1). Because hypermethylation around this CTCF‐binding site has been reported as a possible cause of *GAD1* overexpression,^5^ we further assessed the methylation status of cg15126544 and *GAD1* mRNA expression levels.

### 3.2 Correlation between GAD1 expression and CGI methylation in LADC clinical cases

The DNA methylation status and mRNA expression status were investigated in our panel of LADC tumorous and paired non‐tumorous tissues (Table S2) using pyrosequence‐based methylation assays and rqRT‐PCR‐based expression analysis, respectively. Of the 33 sample sets, 26 (78.8%) demonstrated significantly higher methylation levels in tumor samples than in non‐tumorous tissues (Figure 1C). In the same cases, the mean *GAD1* mRNA expression levels in the tumors were significantly higher than those in the paired non‐tumorous tissues (Figure 1D). There was a slightly positive (*ρ* = 0.251) but significant correlation between methylation levels at cg15126544 and *GAD1* mRNA expression (Figure 1E). The LADC sample set containing 18‐paired samples obtained from TCGA demonstrated similar results both in methylation levels at cg15126544 and *GAD1* mRNA expression (Figure 1F,G and Figure S1). A significant and highly positive correlation between them was also observed in TCGA dataset (*ρ* = 0.706, Figure 1H). Because the gene expression status of cancer cells directly affects their phenotypes, including malignant features, we focused on GAD1 expression in tumors to further assess its prognostic significance in patients with LADC.

### 3.3 Association of GAD1 mRNA expression levels with prognosis in LADC tumors

**Figure 2:**
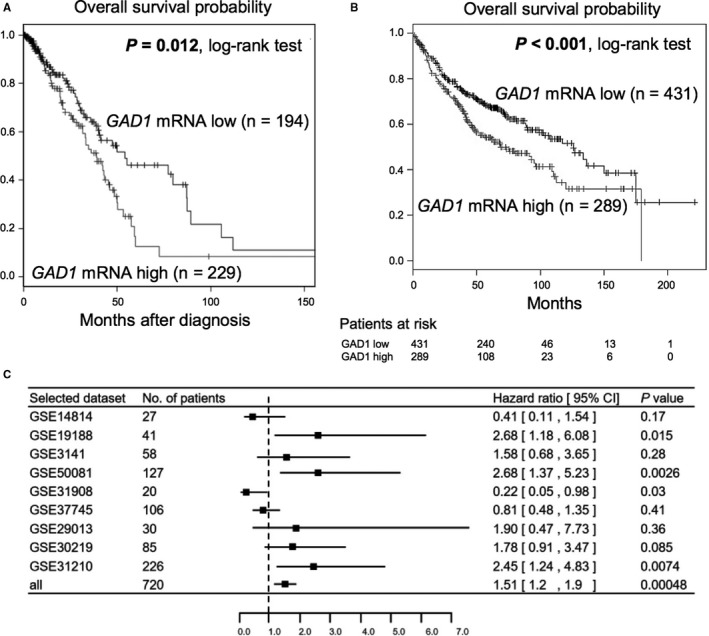
Publicly available datasets showing association between *GAD1* mRNA expression status and prognosis in patients with LADC. (A) Kaplan‐Meier curve for OS rate of 423 LADC patients according to *GAD1* mRNA expression levels using data obtained from the TCGA dataset. *P*‐values were calculated using the log‐rank test. Statistically significant *P*‐values are in boldface type. (B) Kaplan‐Meier curve for OS rate of 720 LADC patients in cohorts GSE14814, GSE19188, GSE3141, GSE50081, GSE31908, GSE37745, GSE29013, GSE30219, and GSE31210 according to *GAD1* mRNA expression levels obtained from the online survival analysis software, Kaplan‐Meier plotter (KM plotter; http://www.kmplot.com). *P*‐values were calculated using the log‐rank test. Statistically significant *P*‐values are in boldface type. (C) Subgroup analysis of KM plotter databases for *GAD1* mRNA expression in LADC. Hazard ratios (HR, center of the box) and 95% confidence intervals (CI, horizontal line) were calculated with Cox's regression models

In our LADC cohort, a sufficient number of cases with high‐quality RNA suitable for expression analysis was not available for survival analysis. Therefore, to test the association between *GAD1* mRNA expression levels in tumors and patients’ prognosis, we first performed survival analysis of 423 patients with LADC using data obtained from TCGA dataset. The OS rate of patients with LADC with higher *GAD1* mRNA expression in tumors was significantly poorer than that of patients with lower *GAD1* mRNA expression in tumors (Figure 2A). Univariate Cox regression analysis using data obtained from TCGA dataset confirmed that high *GAD1* mRNA expression was associated with a worse prognostic significance for OS (Table 1). In multivariate Cox regression analysis, high *GAD1* mRNA expression was also significantly associated with a poorer OS rate, suggesting that *GAD1* mRNA expression is an independent prognostic factor for OS (*P* = 0.036, Table 1).

**Table 1:** Cox proportional hazard regression analysis of overall survival in 400 patients with LADC in TCGA dataset

Factor (number)	Univariate			Multivariate		
	Hazard ratio	95% confidence interval	*P*‐value	Hazard ratio	95% confidence interval	*P*‐value
Sex Male (n = 184) vs. Female (n = 216)	1.048	0.704‐1.560	0.818	1.087	0.705‐1.675	0.706
Age (years) >67 (n = 210) vs. ≤67 (n = 190)	1.348	0.897‐2.025	0.151	1.639	1.079‐2.490	**0.021**
Smoking history Positive (n = 339) vs. Negative (n = 61)	1.069	0.569‐2.006	0.836	1.521	0.766‐3.020	0.230
Pathological stage II, III, IV (n = 184) vs. I (n = 216)	2.620	1.725‐3.979	**6.21E‐6**	—	—	—
Tumor size pT2‐4 (n = 272) vs. pT1 (n = 128)	1.631	0.978‐2.720	0.0609	1.565	0.922‐2.658	0.097
N stage (pN) pN1‐3 (n = 136) vs. pN0 (n = 264)	2.475	1.662‐3.688	**8.32E‐6**	2.487	1.649‐3.750	**1.38E‐5**
M stage (pM) pM1 (n 19) vs. pM0 (n = 381)	1.539	0.773‐3.066	0.220	1.528	0.752‐ 3.103	0.241
*GAD1* mRNA expression High (n = 217) vs. Low (n = 183)	1.749	1.165‐2.626	**6.97E‐3**	1.573	1.029‐2.404	**0.036**

Statistically significant values are in boldface type.

The analysis was performed in 400 patients with complete clinical information in the TCGA dataset.

The population was divided using the auto‐selected best value of *GAD1* mRNA expression as the cutoff.

To validate this result, we performed survival analysis by drawing Kaplan‐Meier survival curves using KM plotter (Figure 2B). A total of 9 studies from the GEO dataset were included (Table S5). In a total of 720 patients with LADC from 9 cohorts, high *GAD1* mRNA expression also significantly correlated with worse OS. In subgroup analysis of OS using datasets of KM plotter, heterogeneous results were obtained among different cohorts. Larger cohorts such as GSE31210 and GSE50081 consistently showed that higher *GAD1* mRNA expression was a poor prognostic factor, whereas cohorts with a smaller number of cases showed varying results (Figure S2). The results of univariate Cox regression analysis confirmed these results (Figure 2C).

### 3.4 Immunohistochemical staining pattern of GAD1 and its association with prognosis in LADC tumors

**Figure 3:**
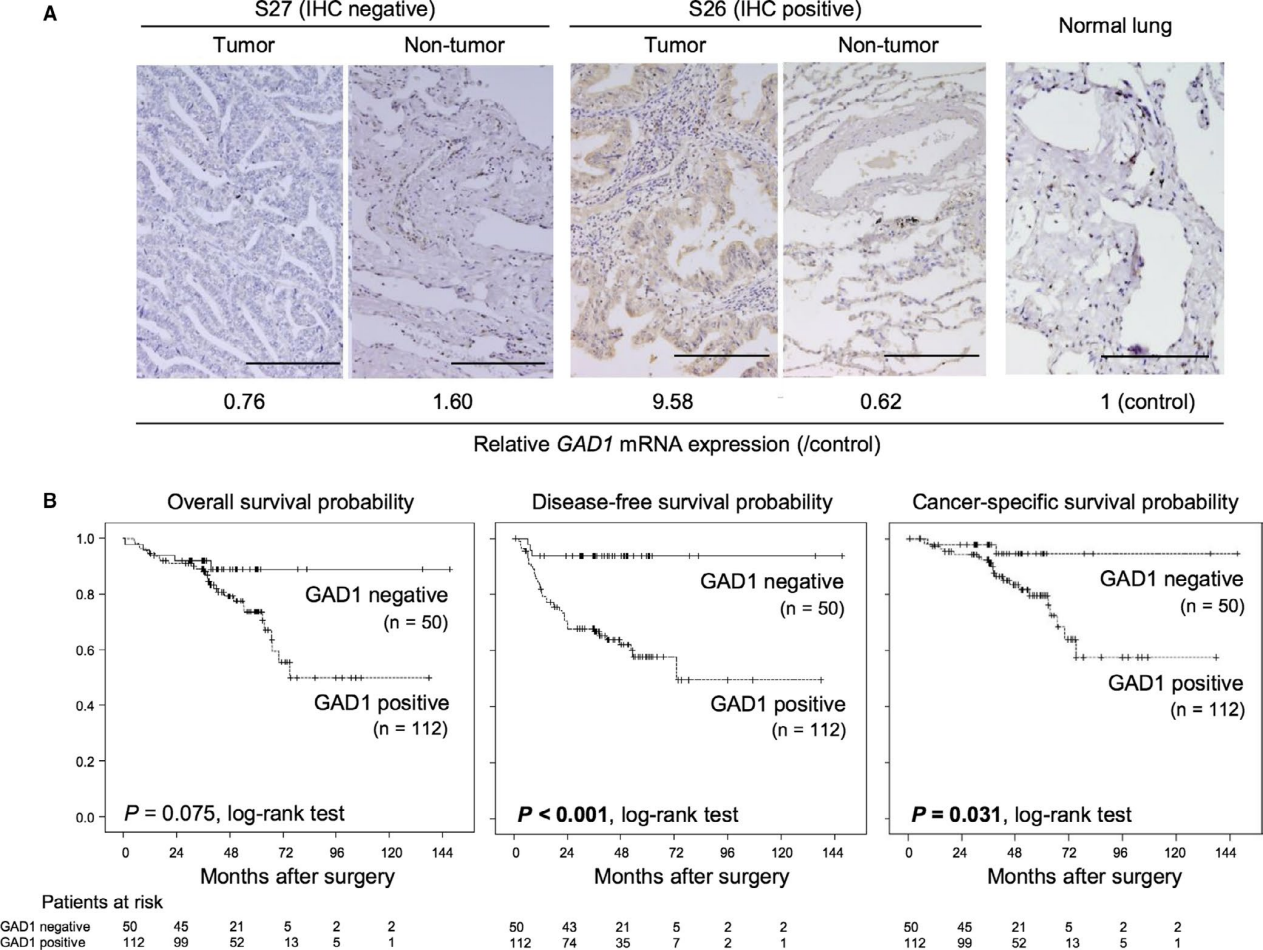
Association between GAD1 protein expression status and prognosis in patients with LADC. (A) Representative images of immunohistochemically detected GAD1 protein in tumors and non‐tumorous lesions of LADC samples and normal lung tissue. Scale bars, 200 μm. The relative GAD1 mRNA expression level of each sample as determined by rqRT‐PCR is also shown. (B) Kaplan‐Meier curves for overall survival, disease‐free survival, and cancer‐specific survival rates of 162 LADC patients according to the immunoreactivity of GAD1. *P*‐values were calculated using the log‐rank test. Statistically significant *P*‐values are in boldface type

To further validate the prognostic significance of GAD1 expression status, we further examined the correlation between GAD1 protein expression and clinicopathological features including prognosis in patients with LADC. We performed immunohistochemical staining of GAD1 in tissue samples from our cohort of 162 patients with LADC (Table S3). Cytoplasmic GAD1 staining was observed in LADC tumor cells with higher mRNA expression, whereas nearly no staining was observed in normal lung epithelial cells and either tumorous or non‐tumorous epithelial cells in LADC with lower mRNA expression (Figure 3A). According to the staining score (Table S6B), 112 patients (69.1%) were classified into the group with tumors showing GAD1 protein overexpression (positive GAD1 immunoreactivity). Among the various clinicopathological factors, the pathological stage, pleural invasion, and lymph vessel invasion were identified as factors significantly and positively associated with positive GAD1 immunoreactivity (Table 2). Lymph node metastasis also tended to be more frequently observed in the positive GAD1 immunoreactivity group.

**Table 2:** Correlation between GAD1 immunoreactivity and clinicopathological factors in 162 patients with LADC

Factor	GAD1 immunoreactivity (n = 162)		*P*‐value^a^
	Negative (n = 50)	Positive (n = 112)	
Male/Female	26/24	55/57	0.865
Age^b^	69.0 ± 9.6	67.4 ± 9.0	0.386
Smoking history^c^ (+/−)	22/27	55/56	0.603
Brinkman index^b^, ^c^	406.5 ± 536.4	485.0 ± 622.7	0.461
Tumor size^b^, ^c^	23.5 ± 14.3	26.1 ± 13.4	0.226
pStage (I/II + III)	39/11	65/47	**0.021**
Lymph node metastasis (±)	8/42	36/76	0.054
Pleural invasion^c^ (±)	5/40	35/72	**0.005**
Vascular invasion^c^ (±)	5/40	22/79	0.284
Lymph vessel invasion^c^(±)	6/39	31/66	**0.023**
*EGFR* mutation^c^(±)	10/6	30/29	0.573
Predominant histologic subtype (lepidic/papillary/acinar/ solid/ enteric)	23/18/4/4/1	36/47/24/5/0	0.068

a *P*‐values were calculated using Fischer's exact test for gender, smoking history, lymph node metastasis, pleural invasion, lymph vessel invasion, and vascular invasion, *EGFR* mutation, and using Student's *t* test for age, Brinkman index, and tumor size and using *χ* ^2^ test for trend for predominant histologic subtype. Statistically significant values (*P* < 0.05) are in boldface type.

b Age, Brinkman index, and tumor size are expressed as the mean ± SD.

c Data of these factors were not available for all patients.

According to the GAD1 protein expression status of LADC tumors, Kaplan‐Meier curves of estimated OS, disease‐free survival (DFS), and cancer‐specific survival (CSS) were generated. Patients with GAD1 protein‐overexpressing tumors showed significantly poorer DFS (*P* < 0.001, log‐rank test) and CSS (*P* = 0.031, log‐rank test) than those without GAD1 protein overexpressing tumors. Patients with GAD1 protein‐overexpressing tumors tended to show poorer OS, although the difference between groups was not significant (Figure 3B). Univariate Cox regression analysis confirmed that positive GAD1 immunoreactivity was significantly associated with a worse prognostic significance for DFS (Table 3). Multivariate Cox regression analysis in 162 patients revealed that GAD1 immunoreactivity was an independent prognostic factor for DFS (*P* = 0.011, hazard ratio = 6.424, Table 3), but not for OS and CSS (Tables S8 and S9).

**Table 3:** Cox proportional hazard regression analysis for disease‐free survival in 162 patients with LADC

Factor	Univariate			Multivariate		
	Hazard ratio	95% confidence interval	*P‐*value	Hazard ratio	95% confidence interval	*P*‐value
Sex Male (n = 81) vs. Female (n = 81)	1.202	0.666‐2.170	0.541	2.459	0.520‐11.624	0.256
Age (years) >67 (n = 87) vs. ≤67 (n = 75)	1.048	0.582‐1.887	0.875	0.995	0.515‐1.922	0.988
Smoking history^a^ Positive (n = 77) vs. Negative (n = 83)	1.302	0.724‐2.344	0.378	0.324	0.068‐1.546	0.158
Pathological stage II, III (n = 58) vs. I (n = 104)	7.466	3.769‐14.789	**<0.001**	—	—	—
Tumor size^a^ pT2‐4 (n = 39) vs. pT1 (n = 115)	2.309	1.241‐4.296	**0.008**	2.033	0.961‐4.303	0.070
N stage (pN) pN1‐3 (n = 44) vs. pN0 (n = 118)	7.100	3.837‐13.140	**<0.001**	2.507	1.057‐5.949	**0.037**
Pleural invasion^a^ Positive (n = 40) vs. Negative (n = 112)	4.926	2.637‐9.202	**<0.001**	2.091	0.977‐4.478	0.058
Vascular invasion^a^ Positive (n = 27) vs. Negative (n = 119)	4.706	2.529‐8.757	**<0.001**	1.139	0.389‐3.341	0.812
Lymph vessel invasion^a^ Positive (n = 37) vs. Negative (n = 105)	5.346	2.809‐10.175	**<0.001**	1.355	0.478‐3.847	0.568
Adjuvant chemotherapy^a^ With (n = 47) vs. Without (n = 106)	2.972	1.614‐5.470	**<0.001**	—	—	—
*EGFR* mutation^a^ Negative (n = 35) vs. Positive (n = 40)	1.285	0.678‐2.433	0.442	—	—	—
Predominant subtype Non‐lepidic (n = 103) vs. Lepidic (n = 59)	6.711	2.392‐ 18.868	**<0.001**	2.725	0.861‐8.621	0.088
GAD1 immunoreactivity Positive (n = 112) vs. Negative (n = 50)	9.341	2.248‐38.824	**0.002**	6.424	1.522‐27.108	**0.011**

Statistically significant values are in boldface type.

a Data of these factors were not available for all patients.

## 4 DISCUSSION

In the present study, we focused on *GAD1* as a hypermethylated gene at specific CpG sites in LADC tumors and demonstrated its overexpression in tumor‐specific and methylation level‐associated manners in LADC. We also demonstrated the prognostic significance of GAD1 mRNA and protein expression levels in resected LADC tumors using various independent publicly available datasets and our cohort, respectively. Our study suggested that GAD1 overexpression may be a useful biomarker for predicting the prognosis of patients with LADC.

GAD1 is known to catalyze the production of *γ*‐aminobutyric acid (GABA) from L‐glutamic acid, the principal inhibitory neurotransmitter in the brain.^13^, ^14^ GAD1 overexpression has been reported in various neoplastic tissues, but not in LADC. Moreover, the associations between clinicopathological characteristics and GAD1 expression have not been well‐established. The most striking finding in this study is the prognostic significance of GAD1 mRNA and protein expression in patients with LADC. Although a sufficient number of RNA samples suitable for expression analysis was not available in our cohort for survival analyses, we used various publicly available data and demonstrated that *GAD1* mRNA overexpression in tumors was significantly associated with poor prognosis (OS) in independent TCGA and GEO datasets of LADC cases. In immunohistochemical analysis using our cohort, a positive cytoplasmic GAD1 staining pattern in tumor cells was significantly associated with poor prognosis, particularly DFS but not OS, in patients with LADC. Although the difference in the association between GAD1 expression and OS among datasets remains unclear, it may be explained by (a) variations in GAD1 mRNA and protein expression, (b) the smaller size of the cohort for immunohistochemical analysis compared to those of cohorts used for mRNA analysis used in our study, and (c) variations in GAD1 expression level and/or pattern among different ethnicities.

Our study also demonstrated that GAD1 protein expression in LADC was significantly associated with pleural invasion and lymph vessel invasion. These findings suggest that GAD1 overexpression might be closely associated with cellular invasion. This hypothesis is supported by previous reports of other cancers. Kimura et al^6^ demonstrated that *GAD1* promotes the cancer cell invasion and metastasis of oral cancer by inducing the nuclear translocation of *β*‐catenin and secretion of MMP7,^15^, ^16^, ^17^, ^18^, ^19^, ^20^ although the regulatory mechanisms of GAD1 in *β*‐catenin translocation remain unclear. In a brain metastasis model, it was reported that the metastatic activity of tumor cells depends on the GAD1‐GABA synthesis pathway.^21^ Further studies are needed to clarify the tumor‐promoting activity of overexpressed GAD1.

Recently, Yan et al^5^ reported hypermethylation‐associated *GAD1* overexpression in colorectal and liver cancers and found that this paradoxical effect was caused by the hypermethylation of the CTCF‐binding site within *GAD1*, which may prevent CTCF binding, inhibit CTCF‐mediated repressive Polycomb repressive complex 2 (PRC2) complex recruitment to the *GAD1* promoter, inhibit PRC2‐induced trimethylation of histone H3 lysine 27 (H3K27m3), and eliminate the blocking activity H3K27m3 for *GAD1* transcription.^22^, ^23^ These observations are contradictory to the well‐established paradigm that promoter DNA methylation represses transcription by inhibiting transcription factor binding and/or chromatin structure modification.^24^, ^25^, ^26^ In this study, we also detected hypermethylation at cg15126544 within the CTCF‐binding site in LADC tumors, and tumor‐specific *GAD1* overexpression was positively associated with hypermethylation at cg15126544 in our cohort and the TCGA dataset. Therefore, methylation of CTCF‐binding sites may regulate *GAD1* expression in LADC as well. However, it remains unknown whether the methylation of CGI or each CpG site around *GAD1*, particularly cg15126544, is the only mechanism underlying the regulation of its transcription. Interestingly, in brain metastatic tumor cells, it was reported that the downregulation of the DNA methyltransferase DNMT1 induced by the brain microenvironment‐derived clusterin resulted in decreased GAD1 promoter methylation and subsequent upregulation of GAD1 expression.^21^ Therefore, even the effect of methylation levels of CpG sites around *GAD1* on its expression level may vary under different conditions or in different cell lineages. Indeed, MethSurv, a web tool for multivariable survival analysis using DNA methylation data obtained from TCGA datasets (https://biit.cs.ut.ee/methsurv/), failed to show the prognostic significance of CpG sites around *GAD1*, including cg15126544 for OS (data not shown). Therefore, the methylation status of some CpG sites around *GAD1* may contribute to its gene expression at some stages of LADC development, but not to the progression of this tumor. The *GAD1* mRNA expression level data in normal lung tissues available in public databases, such as the NIH Genotype‐Tissue Expression Project (https://www.gtexportal.org/), as well as our immunohistochemical staining results revealed no or low GAD1 expression in normal lung tissue, suggesting that GAD1 is specifically expressed in tumor cells and contributes to the progression of tumors in LADC. Because the gene expression status appears to more directly contribute to the establishment of clinicopathological phenotypes in tumor cells, it is necessary to investigate the detailed regulatory mechanisms of GAD1 expression in LADC cells at each developmental stage of the tumor.

There are some limitations to this study. First, we demonstrated the prognostic impact of GAD1 mRNA and protein statuses mainly in Caucasian and Japanese (Asian) populations, respectively, but no data are available to directly compare GAD1 mRNA and protein expression levels among different ethnicities. Because it has been reported that the frequency of acquired alterations, such as epidermal growth factor receptor mutation, in lung tumors can vary across different ethnicities,^27^, ^28^, ^29^ it is possible that the GAD1 expression pattern and/or levels differ between Caucasian and Asian populations. However, the prognostic significance of the *GAD1* mRNA expression status in Japanese cases with LADC was demonstrated by GSE31210 in GEO datasets (Figure 2C and Figure S2). Meta‐analysis using 9 GEO datasets, including GSE31210 and 8 other studies from western countries (Table S5) also revealed the prognostic significance of the GAD1 mRNA expression status (Figure 2C), suggesting that GAD1 overexpression is a common prognostic factor in various populations. Second, our patient cohort was relatively small even for immunohistochemical analysis, and a sufficient number of samples were not available for mRNA expression analysis to perform survival analysis. Prospective multiinstitutional studies are needed to further validate the prognostic value of GAD1 overexpression in patients with LADC.

## 5 CONCLUSION

GAD1 overexpression appears to be a significant and independent prognostic indicator in patients with resected LADC at both the mRNA and protein levels. This information may be helpful for identifying patients at high risk of recurrence and overall survival after tumor resection of LADC.

## CONFLICT OF INTEREST

All authors declare no conflicts of interest associated with this manuscript.
