# Supplementary figures and images for: Prognostic significance of GAD1 overexpression in patients with resected lung adenocarcinoma
Source: Cancer Med. 2019 Jun 17;8(9):4189–99. doi: 10.1002/cam4.2345 (PMC6675743; doi:10.1002/cam4.2345)

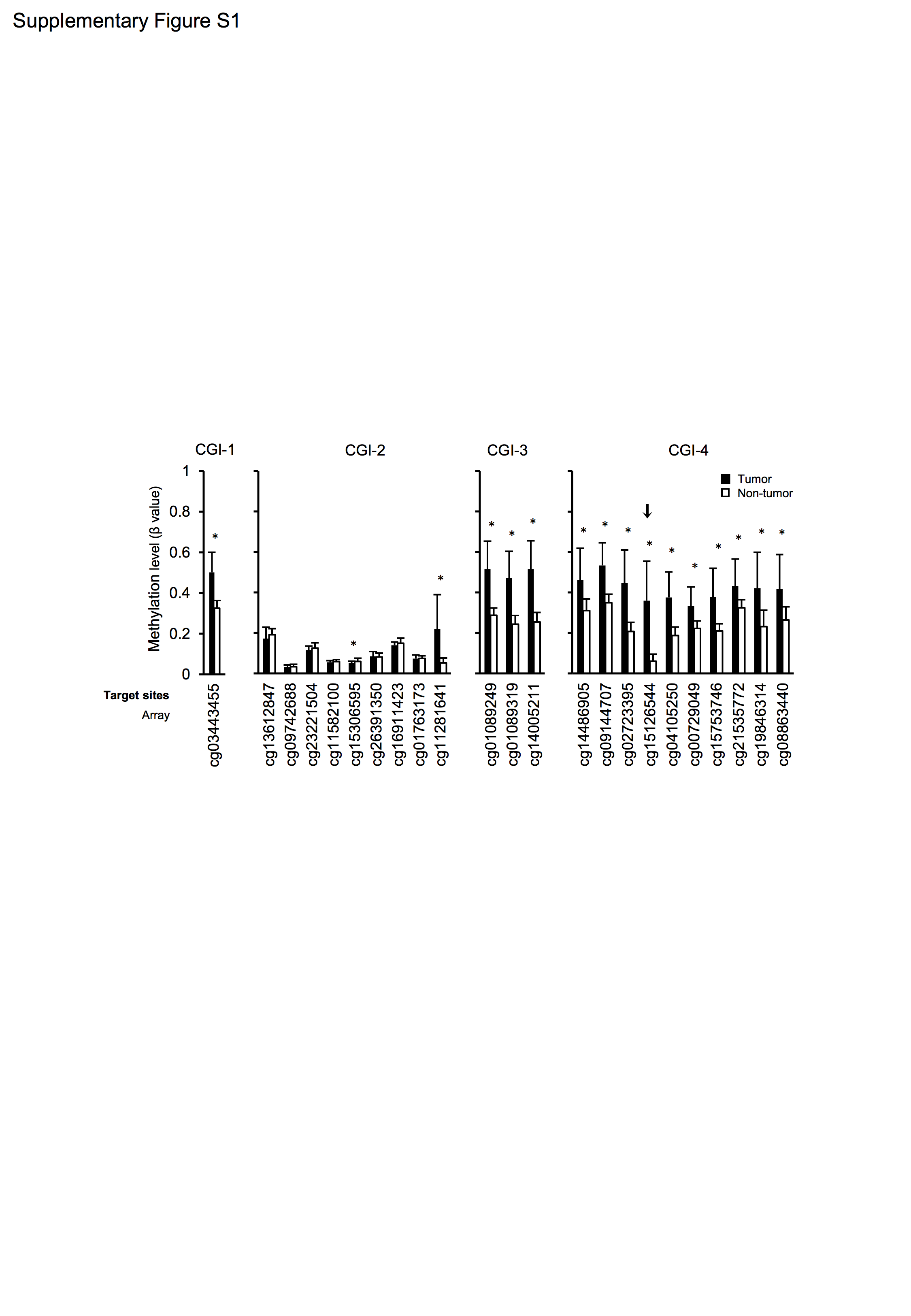

Supplement: Supplementary file 1 [file CAM4-8-4189-s001.tiff]

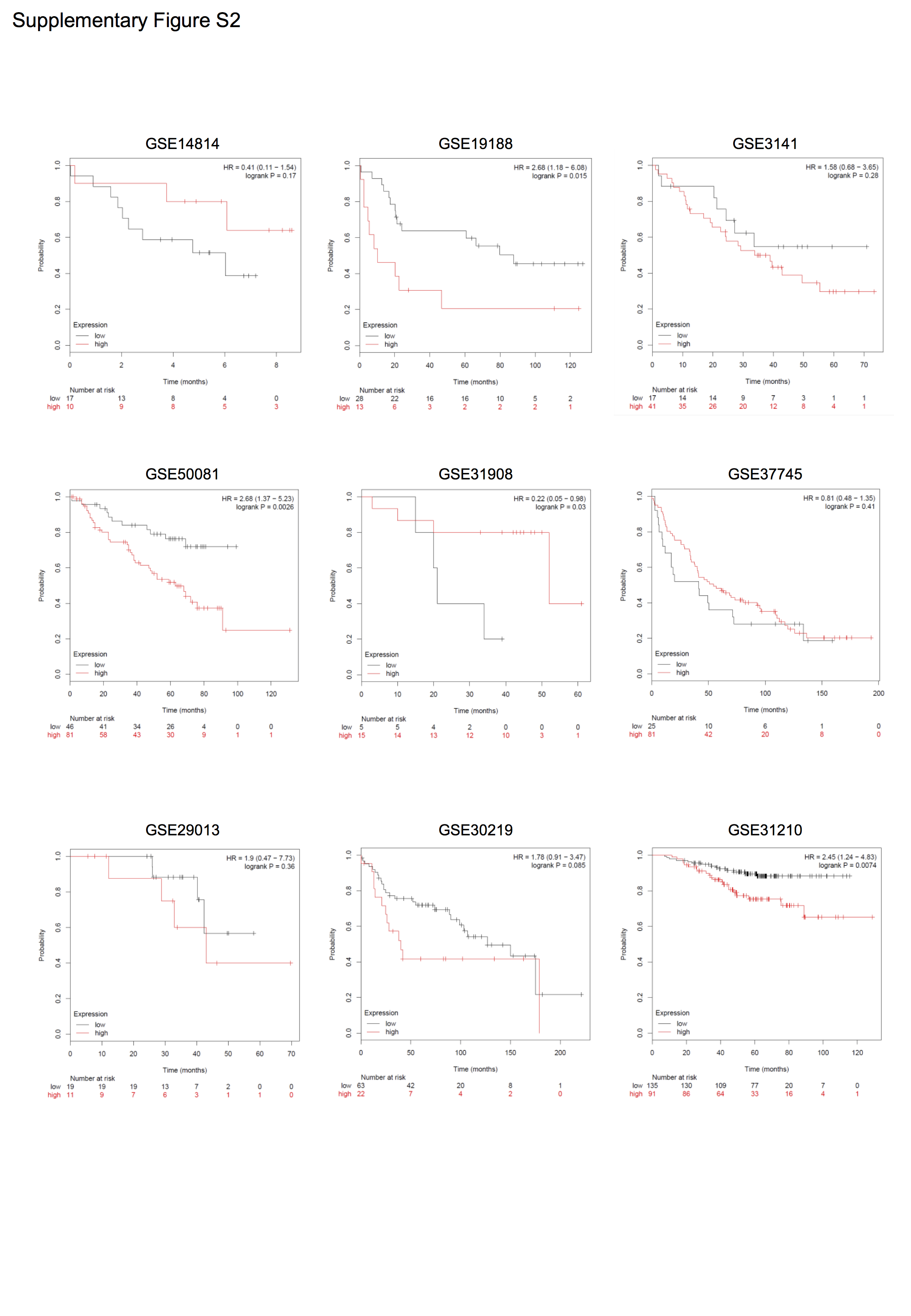

Supplement: Supplementary file 2 [file CAM4-8-4189-s002.tiff]
