# Supplementary material for: Prognostic significance of GAD1 overexpression in patients with resected lung adenocarcinoma
Source: Cancer Med. 2019 Jun 17;8(9):4189–99. doi: 10.1002/cam4.2345 (PMC6675743; doi:10.1002/cam4.2345)
Supplement: Supplementary file 3 [file CAM4-8-4189-s003.docx]

Supplementary Table S1. The top 14 CpG islands significantly hypermethylated in tumorous tissues of 12 stage-I LADC cases^4^

| No. | CpG island | Adjusted *P*-value^a^ | β-difference^b^ | Gene name |
| --- | --- | --- | --- | --- |
| 1 | chr7:153583317-153585666 | 0.000495704 | 0.277562652 | *DPP6* |
| 2 | chr19:52390841-52391368 | 0.000671077 | 0.291495741 | *ZNF577* |
| 3 | chr11:125774292-125774584 | 0.000839411 | 0.271871389 | *DDX25* |
| 4 | chr3:62355315-62355534 | 0.001348289 | 0.25890625 | *FEZF2* |
| 5 | chr1:156863415-156863711 | 0.001564128 | 0.369176667 | *PEAR1* |
| 6 | chr15:37390175-37390380 | 0.002225792 | 0.324917222 | *MEIS2* |
| 7 | chr1:248020330-248021252 | 0.00443318 | 0.270335 | *TRIM58* |
| 8 | chr12:103696090-103696418 | 0.006552561 | 0.318931667 | *C12orf42* |
| 9 | chr7:158110569-158110881 | 0.008975336 | 0.270233333 | *PTPRN2* |
| 10 | chr6:50810642-50810994 | 0.010799023 | 0.30752125 | *TFAP2B* |
| 11 | chr5:134363092-134365146 | 0.011483039 | 0.262798796 | *PITX1* |
| 12 | chr19:58545115-58545897 | 0.011599292 | 0.27722213 | *ZSCAN1* |
| 13 | chr6:50791110-50791573 | 0.012733507 | 0.331794167 | *TFAP2B* |
| **14** | **chr2:171676552-171676980** | **0.017464759** | **0.251871944** | ***GAD1*** |

The row corresponding to *GAD1* is in boldface type.

^a^Differences between methylation levels (β-values) of CpG islands in tumors and paired non-tumorous tissues were assessed by paired *t*-test. *P*-values were adjusted with the Benjamini–Hochberg correction (false discovery rate, FDR). CpG islands were sorted by the adjusted *P*-value.

^b^β-differences (differential methylation levels) represent the average of [(β-value of tumorous tissue) - (β-value of paired non-tumorous tissue)] in 12 stage-I LADC cases.

Supplementary Table S2. Clinicopathological characteristics of 33 patients with LADC analyzed by qPCR and pyrosequencing

| Characteristics | Number |
| --- | --- |
| Gender  Male  Female | 18  15 |
| Age (years) | 62.9 ± 9.6 |
| Stage  Ia, Ib  IIa, IIb  IIIa, IIIb | 16  8  9 |
| Smoking History  +  - | 15  18 |
| Brinkman Index | 616.7 ± 745.4 |

Age and Brinkman index are expressed as the mean ± standard deviation.

Supplementary Table S3. Characteristics of 162 patients with LADC analyzed by immunohistochemistry

| Characteristics | N = 162 (%) |
| --- | --- |
| Gender  Male  Female | 81 (50.0%)  81 (50.0%) |
| Age (years) | 67.0 ± 9.2 |
| Stage  Ia, Ib  IIa, IIb  IIIa, IIIb | 104 (64.2%)  26 (16.0%)  32 (19.8%) |
| *EGFR* mutation  　　　　Positive  Negative  　　　 Unknown | 40 (24.7%)  35 (21.6%)  87 (53.7%) |
| Predominant histologic subtype  lepidic  papillary  acinar  solid  enteric | 59 (36.4%)  65 (40.1%)  28 (17.3%)  9 (5.6%)  1 (0.6%) |
| Adjuvant chemotherapy  With  Without  Unknown | 47 (29.0%)  106 (65.4%)  9 (5.6%) |
| Smoking History  +  -  Unknown | 78 (48.1%)  82 (50.6%)  2 (1.2%) |
| Brinkman Index | 461.0 ± 597.0 |

Age and Brinkman index are expressed as the mean ± standard deviation.

Supplementary Table S4. List of primer sets used in qPCR and pyrosequencing

|  | Gene/primer name |  | Sequence/ID |
| --- | --- | --- | --- |
| **TaqMan gene expression assay** | | | |
|  | *GAD1* | FAM | Hs01065893_m1 |
|  | *GAPDH* | FAM | Hs02758991_g1 |
|  |  |  |  |
| **Pyrosequencing of *GAD1*** | | | |
|  | cg15126544 | Forward | 5'-TGGTTTTTAGGGGTTTTTTTTTTTGGA-3' |
|  |  | Reverse | 5'-ACAAATACACCCCCTTTAATCTACTCTCC-3' |
|  |  | Sequence | 5'-GTAGAAGAGGGAGGAA-3' |

Supplementary Table S5. List of GEO data sets

| GEO accession | Survival period | Submission  date | Number of patients | Country | | Race | | Platform | |  |  |  |
| --- | --- | --- | --- | --- | --- | --- | --- | --- | --- | --- | --- | --- |
| GSE14814 | from date of random assignment to death from disease or treatment complication | 12-Feb-9 | 27 | Canada USA  Germany | | NA | | HG-U133A, | |  |  |  |
| GSE19188 | NA | 25-Nov-9 | 41 | Netherlands | | Mostly Caucasian | | HG-U133_Plus_2 | |  |  |  |
| GSE3141 | NA | 16-Aug-5 | 58 | USA | | NA | | HG-U133_Plus_2 | |  |  |  |
| GSE50081 | NA | 21-Aug-13 | 127 | Canada | | NA | | HG-U133_Plus_2 | |  |  |  |
| GSE31908 | NA | 6-Sep-11 | 20 | USA | | Mostly  Caucasian | | HG-U133A HG-U133B HG-U133_Plus_2 | |  |  |  |
| GSE37745 | NA | 3-May-12 | 106 | Sweden | | NA | | HG-U133_Plus_2 | |  |  |  |
| GSE29013 | from the date of surgery to death or the last follow-up contact. | 2-May-11 | 30 | USA | | Mostly  Caucasian | | HG-U133_Plus_2 | |  |  |  |
| GSE30219 | NA | 26-Jun-11 | 85 | France  USA | | NA | | HG-U133_Plus_2 | |  |  |  |
| GSE31210 | NA | 4-Aug-11 | 226 | Japan | | Asian | | HG-U133_Plus_2 | |  |  |  |
|  | | | | |  | |  | |  | |  |  |

Supplementary Table S6. Evaluation criteria for GAD1 immunohistochemistry

1. Proportion and intensity scores for GAD1 staining in immunohistochemical analysis

| Proportion score (PS) | |  | Intensity score (IS) | |
| --- | --- | --- | --- | --- |
| Score | Observation |  | Score | Observation |
| 1 | < 25% |  | 0 | None |
| 2 | 26 - 50% |  | 1 | Weak |
| 3 | 51 - 75% |  | 2 | Intermediate |
| 4 | 76% ≤ |  | 3 | Strong |

1. Evaluation of GAD1 immunoreactivity using PS and IS

| GAD1 immunoreactivity | Sum of PS and IS | Number of cases |
| --- | --- | --- |
| Negative | 1 | 0 |
|  | 2 | 14 |
|  | 3 | 10 |
|  | 4 | 26 |
| Positive | 5 | 44 |
|  | 6 | 43 |
|  | 7 | 25 |

The staining score is defined as the sum of the proportion and intensity scores.

A staining score ≥ 5 indicated overexpression of the GAD1 protein (positive GAD1 immunoreactivity).

Supplementary Table S7. The methylation levels of each CpG site of *GAD1* in tumorous and non-tumorous samples

| CpG site | β-value (average^a^) | |  | β-value (SD^b^) | | *P*-value^c^ | β-difference^d^ |
| --- | --- | --- | --- | --- | --- | --- | --- |
|  | Tumor | Non-tumor |  | Tumor | Non-tumor |  |  |
| cg09404592 | 0.109475 | 0.0853917 |  | 0.04619235 | 0.02516931 | 0.1810443145 | 0.024083333 |
| cg03443455 | 0.462661667 | 0.3074692 |  | 0.09759064 | 0.04014665 | 0.0004807189 | 0.1551925 |
| cg00782607 | 0.106084167 | 0.0710583 |  | 0.04960498 | 0.03644913 | 0.0389929681 | 0.035025833 |
| cg13612847 | 0.133873333 | 0.1472975 |  | 0.03817365 | 0.03253079 | 0.0972520412 | -0.013424167 |
| cg03448612 | 0.083106667 | 0.0758158 |  | 0.02049695 | 0.03057835 | 0.3611596712 | 0.007290833 |
| cg09742688 | 0.019295 | 0.014535 |  | 0.00964859 | 0.00580472 | 0.1767899083 | 0.00476 |
| cg23221504 | 0.100365 | 0.1095158 |  | 0.02678416 | 0.03834864 | 0.3202119878 | -0.009150833 |
| cg00915206 | 0.067003333 | 0.0671983 |  | 0.01743706 | 0.03309326 | 0.9850457390 | -0.000195 |
| cg11582100 | 0.05327 | 0.0542092 |  | 0.01019424 | 0.02677933 | 0.8871008599 | -0.000939167 |
| cg15306595 | 0.0394325 | 0.0438158 |  | 0.01045799 | 0.01931569 | 0.4524979803 | -0.004383333 |
| cg19538089 | 0.104192727 | 0.0801767 |  | 0.05266865 | 0.02819045 | 0.1330389745 | 0.024016061 |
| cg26391350 | 0.086990833 | 0.0757083 |  | 0.02871752 | 0.03569546 | 0.2703867579 | 0.0112825 |
| cg16911423 | 0.179124167 | 0.13477 |  | 0.05271661 | 0.02415412 | 0.0169907853 | 0.044354167 |
| cg01763173 | 0.085408333 | 0.0639708 |  | 0.03334425 | 0.02293707 | 0.0705795610 | 0.0214375 |
| cg11281641 | 0.154460833 | 0.0533167 |  | 0.0959479 | 0.02516992 | 0.0046353604 | 0.101144167 |
| cg07420274 | 0.536216667 | 0.3580283 |  | 0.06408349 | 0.05326207 | 0.0001252001 | 0.178188333 |
| cg01089249 | 0.529403333 | 0.2895042 |  | 0.06934644 | 0.02343744 | 0.0000006700 | 0.239899167 |
| cg01089319 | 0.505844167 | 0.256055 |  | 0.06506377 | 0.03497551 | 0.0000011299 | 0.249789167 |
| cg14005211 | 0.539773333 | 0.2738458 |  | 0.0827698 | 0.05432622 | 0.0000013853 | 0.2659275 |
| cg14486905 | 0.46974 | 0.2449983 |  | 0.12168293 | 0.04163571 | 0.0002223438 | 0.224741667 |
| cg09144707 | 0.494621667 | 0.2901625 |  | 0.10770392 | 0.03041366 | 0.0000452368 | 0.204459167 |
| cg02723395 | 0.411985 | 0.1740225 |  | 0.15358608 | 0.0351129 | 0.0005880955 | 0.2379625 |
| **cg15126544** | **0.363693333** | **0.0397042** |  | **0.14160994** | **0.02161663** | **0.0000079194** | **0.323989167** |
| cg04105250 | 0.337811667 | 0.1510408 |  | 0.11281548 | 0.02977583 | 0.0001733147 | 0.186770833 |
| cg00729049 | 0.2934125 | 0.1690383 |  | 0.08912118 | 0.02842007 | 0.0014668716 | 0.124374167 |
| cg15753746 | 0.363454167 | 0.1337517 |  | 0.17759626 | 0.03602908 | 0.0009859990 | 0.2297025 |
| cg21535772 | 0.4300025 | 0.2728233 |  | 0.09775366 | 0.03462184 | 0.0004699895 | 0.157179167 |
| cg19846314 | 0.445076667 | 0.2232308 |  | 0.17797695 | 0.07571523 | 0.0024014465 | 0.221845833 |
| cg08863440 | 0.403660833 | 0.2232942 |  | 0.15063177 | 0.06307915 | 0.0033596932 | 0.180366667 |
| cg07620853 | 0.5666125 | 0.5479283 |  | 0.19697479 | 0.14942316 | 0.7081756486 | 0.018684167 |

The row corresponding to cg15126544 is in **boldface type**.

^a^The average methylation level of 12 LADC samples.

^b^The standard deviation (SD) of methylation levels of 12 LADC samples.

^c^Differences between methylation levels (β-values) of CpG islands in tumors and paired non-tumorous tissues were assessed by paired *t*-test.

^d^β-differences (differential methylation levels) represent the average of [(β-value of tumorous tissue) - (β-value of paired non-tumorous tissue)] in 12 stage-I LADC cases.

Supplementary Table S8. Cox proportional hazard regression analysis of overall survival in 162 patients with LADC

| Factor | Univariate | | |  | Multivariate | | |
| --- | --- | --- | --- | --- | --- | --- | --- |
|  | Hazard ratio | 95% confidence interval | *P-*value |  | Hazard ratio | 95% confidence interval | *P*-value |
| Sex  Male (n=81)  vs. Female (n=81) | 3.219 | 1.452 - 7.138 | **0.004** |  | 1.311 | 0.220 - 7.802 | 1.311 |
| Age (years)  >67 (n=87)  vs. ≤67 (n=75) | 2.471 | 1.169 - 5.224 | **0.018** |  | 2.562 | 1.073 - 6.120 | **0.034** |
| Smoking history^a^  Positive (n=77)  vs. Negative (n=83) | 4.177 | 1.817 - 9.602 | **0.001** |  | 2.166 | 0.341 - 13.759 | 0.413 |
| Pathological stage  II, III (n=58)  vs. I (n=104) | 4.328 | 1.999 - 9.372 | **< 0.001** |  | - | - | - |
| Tumor size^a^  pT2-4 (n=39)  vs. pT1 (n=115) | 2.262 | 1.119 - 4.573 | **0.023** |  | 2.466 | 1.116 - 5.447 | **0.026** |
| N stage (pN)  pN1-3 (n=44)  vs. pN0 (n=118) | 3.577 | 1.789 - 7.151 | **< 0.001** |  | 0.909 | 0.343 - 2.410 | 0.848 |
| Pleural invasion^a^  Positive (n=40)  vs. Negative (n=112) | 2.051 | 0.987 - 4.264 | 0.054 |  | 1.635 | 0.626 - 4.267 | 0.315 |
| Vascular invasion^a^  Positive (n=27)  vs. Negative (n=119) | 2.735 | 1.284 – 5.826 | **0.009** |  | 0.487 | 0.157 - 1.512 | 0.213 |
| Lymph vessel invasion^a^  Positive (n=37)  vs. Negative (n=105) | 4.700 | 2.203 - 10.027 | **< 0.001** |  | 3.897 | 1.311 - 11.580 | **0.014** |
| Adjuvant chemotherapy^a^  With (n=47)  vs. Without (n=106) | 0.996 | 0.472 - 2.101 | 0.991 |  | - | - | - |
| *EGFR* mutation^a^  Negative (n=35)  vs. Positive (n=40) | 2.882 | 1.151 - 2.564 | **0.024** |  | - | - | - |
| Predominant subtype  Non-lepidic (n=103)  vs. Lepidic (n=59) | 3.311 | 1.156- 9.524 | **0.026** |  | 2.841 | 0.590 - 13.699 | 0.193 |
| GAD1 immunoreactivity  Positive (n=112)  vs. Negative (n=50) | 2.315 | 0.895 - 5.992 | 0.084 |  | 1.216 | 0.366 - 4.042 | 0.750 |

Statistically significant values are in boldface type.

^a^Data of these factors were not available for all patients.

Supplementary Table S9. Cox proportional hazard regression analysis of cancer-specific survival in 162 patients with LADC

| Factor | Univariate | | |  | Multivariate | | |
| --- | --- | --- | --- | --- | --- | --- | --- |
|  | Hazard ratio | 95% confidence interval | *P-*value |  | Hazard ratio | 95% confidence interval | *P*-value |
| Sex  Male (n=81)  vs. Female (n=81) | 1.945 | 0.827 - 4.576 | 0.127 |  | 1.160 | 0.194 - 6.923 | 0.871 |
| Age (years)  >67 (n=87)  vs. ≤67 (n=75) | 1.735 | 0.749 - 4.021 | 0.199 |  | 1.932 | 0.748 - 4.987 | 0.173 |
| Smoking history^a^  Positive (n=77)  vs. Negative (n=83) | 2.599 | 1.076 - 6.279 | **0.034** |  | 1.398 | 0.221 - 8.839 | 0.722 |
| Pathological stage  II, III (n=58)  vs. I (n=104) | 7.706 | 2.606 - 22.791 | **< 0.001** |  | - | - | - |
| Tumor size^a^  pT2-4 (n=39)  vs. pT1 (n=115) | 1.994 | 0.847 - 4.697 | 0.114 |  | 1.867 | 0.733 - 4.758 | 0.191 |
| N stage (pN)  pN1-3 (n=44)  vs. pN0 (n=118) | 6.066 | 2.488 - 14.789 | **< 0.001** |  | 1.322 | 0.398 - 4.395 | 0.649 |
| Pleural invasion^a^  Positive (n=40)  vs. Negative (n=112) | 2.331 | 0.957 - 5.677 | 0.063 |  | 1.255 | 0.429 - 3.673 | 0.679 |
| Vascular invasion^a^  Positive (n=27)  vs. Negative (n=119) | 4.089 | 1.697 – 9.854 | 0.002 |  | 0.892 | 0.236 - 3.378 | 0.867 |
| Lymph vessel invasion^a^  Positive (n=37)  vs. Negative (n=105) | 5.610 | 2.239 - 14.055 | **< 0.001** |  | 2.654 | 0.703 - 10.022 | 0.150 |
| Adjuvant chemotherapy^a^  With (n=47)  vs. Without (n=106) | 1.036 | 0.428 - 2.504 | 0.938 |  | - | - | - |
| *EGFR* mutation^a^  Negative (n=35)  vs. Positive (n=40) | 3.165 | 1.188 - 8.403 | **0.021** |  | - | - | - |
| Predominant subtype  Non-lepidic (n=103)  vs. Lepidic (n=59) | 9.804 | 1.311- 71.429 | **0.026** |  | 3.378 | 0.392 - 29.411 | 0.268 |
| GAD1 immunoreactivity  Positive (n=112)  vs. Negative (n=50) | 4.323 | 1.015 - 18.420 | **0.048** |  | 3.400 | 0.415- 27.827 | 0.254 |

Statistically significant values are in boldface type.

^a^Data of these factors were not available for all patients.
